# Digital Platform for the Prevention of Suicidal Behaviour and Non-Suicidal Self-Injuries in Adolescents: The SmartCrisis-Teen Study Protocol

**DOI:** 10.3390/bs14090740

**Published:** 2024-08-25

**Authors:** Sofía Abascal-Peiró, Inmaculada Peñuelas-Calvo, Adrian Alacreu-Crespo, Pilar Alejandra Sáiz, Alejandro De la Torre-Luque, Miguel Ruiz-Veguilla, María Luisa Barrigón, Philippe Courtet, Jorge López-Castroman, Enrique Baca-García, Alejandro Porras-Segovia

**Affiliations:** 1 Department of Psychiatry, University Hospital Fundación Jiménez Díaz, 28040 Madrid, Spain; sofiaabascalp@gmail.com (S.A.-P.); ebacgar2@yahoo.es (E.B.-G.); 2 Department of Child and Adolescent Psychiatry, University Hospital 12 de Octubre, 28041 Madrid, Spain; inmapenuelas@gmail.com; 3 Department of Psychology and Sociology, Area of Personality, Assessment and Psychological Treatment, University of Zaragoza, 50009 Zaragoza, Spain; alacreu.a@gmail.com; 4 Department of Psychiatry, School of Medcine, University of Oviedo, 33003 Oviedo, Spain; frank@uniovi.es; 5 Department of Legal Medicine, Psychiatry and Pathology, School of Medicine, Universidad Complutense de Madrid, 28040 Madrid, Spain; af.delatorre@ucm.es; 6 Department of Psychiatry, University Hospital Virgen del Rocío, 41013 Sevilla, Spain; npdunit@gmail.com; 7 Institute of Psychiatry and Mental Health, Hospital General Universitario Gregorio Marañón, 28007 Madrid, Spain; marisabe@gmail.com; 8 Department of Emergency Psychiatry and Acute Care, Centre Hospitalier Universitaire Montpellier, University of Montpellier, 34090 Montpellier, France; philippecourtet@gmail.com; 9 Department of Signal Theory and Communications, Universidad Carlos III de Madrid, Leganés, 28903 Madrid, Spain; jorgecastroman@gmail.com; 10 Translational Psychiatry Research Group, Instituto de Investigación Sanitaria Fundación Jiménez Díaz, 28040 Madrid, Spain; 11 CIBERSAM (Centro de Investigación en Salud Mental), Carlos III Institute of Health, 28029 Madrid, Spain; 12 Department of Psychiatry, University Hospital Rey Juan Carlos, Móstoles, 28933 Madrid, Spain; 13 Department of Psychiatry, Universidad Autónoma de Madrid, 28049 Madrid, Spain; 14 Department of Psychiatry, General Hospital of Villalba, Villalba, 28400 Madrid, Spain; 15 Department of Psychiatry, University Hospital Infanta Elena, Valdemoro, 28342 Madrid, Spain

**Keywords:** suicide, suicide ideation, suicide attempt, non-suicidal self-injury, eHealth, mHealth

## Abstract

Suicidal behavior and Non-Suicidal Self-Injuries (NSSIs) are a major health problem in the adolescent population. New technologies can contribute to the development of innovative interventions in suicide prevention. Here, we present the SmartCrisis-Teen study protocol. The study consists of a randomized clinical trial which aims to evaluate the effectiveness of a digital safety plan to prevent suicidal behavior and NSSIs in adolescents. This is a multicentric study which will be conducted among the adolescent population, both in clinical and student settings, with a target sample of 1080 participants. The intervention group will receive an Ecological Momentary Intervention (EMI) consisting of a digital safety plan on their mobile phone. All participants will receive their Treatment As Usual (TAU). Participants will be followed for six months, with weekly and monthly telephone visits and face-to-face visits at three and six months. Participants will be assessed using traditional questionnaires as well as Ecological Momentary Assessment (EMA) and Implicit Association Tests (IATs). With this intervention, we expect a reduction in NSSIs through the acquisition of coping strategies and a decrease in suicidal behavior over the course of follow-up. This study provides a novel, scalable digital intervention for preventing suicidal behavior and NSSIs in adolescents, which could contribute to improving adolescent mental health outcomes globally.

## 1. Introduction

Adolescence is a complex time in life when the risk of developing mental health problems increases. Age-specific risks, the acquisition of social roles, and the search for individuation and connection to peers may lead to social insecurity, feelings of uncertainty and increased influence of the peer group on behavior [1,2]. Some authors suggest that the concept of adolescence should be extended to 18–24 years of age (the range commonly known as young adulthood) because people in this group share neurobiological characteristics with people aged 13–18 [3]. Comparing the psychopathology of both groups could shed light on this debate. While concern about adolescent mental health is by no means a new concept, worries have intensified since the COVID-19 pandemic, as an increase in the range of mental health problems, including a spike in Non-Suicidal Self-Injuries (NSSI), has been reported [4].

Among the many mental health problems that can affect young people, suicidal behavior and Non-Suicidal Self-Injury (NSSI) are receiving growing attention due to the dramatic increase observed in recent years [5]. Suicide is the second leading cause of death among people aged 10–24 years old [6], and suicide attempts impose a significant financial burden on health care systems [7]. In 2000, suicide represented 8.5% of the total deaths in the 15–24 age group in Spain, and in 2021 this percentage raised to 15.8% [8]. With regard to NSSIs, not only does they increase the risk of suicide [9], but it is also associated with other mental health problems, such as emotional dysregulation or substance use [10].

Most research in suicide prevention in adolescents is conducted in clinical settings, providing a crucial look into patients at risk of suicide. In the mid- to long-term, the most important risk factor is a history of suicidal ideation or behavior. Therefore, individuals who previously attempted or considered suicide are at higher risk of suicide-related death [11]. Secondary prevention is thus a crucial area in preventing suicide deaths [12]. However, researching suicidal behavior in school settings is also important, as the rise of NSSIs is particularly alarming in schools. As part of the EPISAM-school study, suicide risk screening was carried out in schools in the community of Madrid, where it was found that 35% of young people had self-harmed, 12.6% were at risk of suicide and 5.7% had attempted suicide [2].

The use of apps for improving mental health problems such as depression [13], substance use [14] or poor sleep [15] is spreading, and they could be particularly useful in the adolescent population, given their high digital literacy. Additionally, smartphone use among adolescents is as high as 95% [16], which makes these devices a powerful tool for improving access to mental health care.

In suicide prevention, apps can be used for risk monitoring and for intervention. Smartphone-based risk monitoring is commonly performed through Ecological Momentary Assessment (EMA), which consists of asking patients daily questions, providing a continuous, real-time source of information collected in the patient’s usual environment [17]. The therapeutic version of EMA is called Ecological Momentary Intervention (EMI), which can be used as a complementary therapeutic approach with continual availability and low cost [18] in a model that allows patients to become involved in their own treatment [19]. One of the evidence-based interventions that can be used in the form of an EMI is the Safety Plan, a set of personalized strategies aimed at dealing with a suicidal crisis [20,21]

The combination of EMA and EMI presents a therapeutic potential in suicide prevention, allowing for the possibility for the intervention to be automatically triggered when a high risk of suicide is detected. 

Another useful digital assessment tool is the Death/Suicide Implicit Association Test (D/S IAT) and the Self-Injury Implicit Association Test (S-I IAT). These tests measure the relative strength of automatic associations related to suicide, death and self-harm [22]. These IATs are brief computer-administered tests that measure reaction times when classifying semantic stimuli. In the case of the D/S IAT and the S-IIAT, the topics under consideration are life and death/suicide and self-injuries, respectively. These tests have shown high predictive power in some studies [22,23,24,25]. Finally, they have the potential to be unconscious in nature, so people have very little control over their answers.

The use of apps for the prevention of suicide behavior in the adolescent population is an area that has received little attention. Jiménez-Muñoz et al. [26] carried out a systematic review focusing on EMI interventions in suicidal behavior prevention, showing that the young population typically exhibit high rates of interest and satisfaction in these tools [27,28,29,30]. For instance, the BeyondNow application, which features a digital safety plan, underwent a successful before-and-after study on a cohort of 20 adolescent females at high risk of suicide [31]. Additionally, the BlueIce app, which concentrates on stopping self-harm, underwent a before-and-after study with a group of 44 adolescents who had a history of self-harm, leading to a decrease in self-harming actions as well as depressive symptoms and anxiety [27].

In general, the studies conducted to date have been carried out with small sample sizes and short follow-up periods. No similar studies have been conducted in Spain. Furthermore, to our knowledge, only one case series [32] and one clinical trial protocol [33] have considered the combination of EMA with EMI. Also, to our knowledge, no previous studies have combined the use of EMA or EMI with IATs. Combining different digital tools can optimize the user experience and contribute to the effectiveness of assessments and interventions. 

The SmartCrisis-Teen protocol is significant, as it offers a scalable, evidence-based digital solution that integrates Ecological Momentary Interventions (EMIs) with real-time monitoring and predictive assessments, aiming to address the growing rates of suicidal behavior and NSSIs in adolescents. This project represents a critical contribution to the field of adolescent mental health by enhancing accessibility and personalizing suicide prevention strategies.

Here, we present the SmartCrisis-Teen study protocol, a Randomized Clinical Trial whose main objective is to evaluate the effectiveness of a digital safety plan combined with EMA to prevent suicidal behavior and NSSIs in the adolescent population. Our secondary objectives are to determine the feasibility and acceptability of the safety plan and to validate the use of the D/S IAT and SI IAT with respect to the gold standard Columbia Suicide Severity Rating Scale (CSSRS) [34]. Our hypothesis, based on previous studies carried out in the adult population [35], is that there will be a significant reduction in suicidal behavior and NSSIs episodes after the follow-up, which will be translated in lower scores in the CSSRS. As a secondary hypothesis, we expect that the digital safety plan will be feasible and well-accepted and that the D/S IAT and SI IAT will demonstrate adequate external and internal validity.

## 2. Materials and Methods

### 2.1. Setting and Design

This study was approved by the Ethics Committee of the University Hospital Fundación Jiménez Díaz. Participants—when they are over 18 years old—or their legal tutors—when they are underaged—will give informed consent to participate in the study. Participants will not receive any financial incentive to become involved in the project.

This is a multicenter randomized clinical trial taking place in various clinical and educational settings across in Spain. Participants will be randomly assigned to either the intervention group, which will receive the digital safety plan on their mobile phone, and a control group. All participants will receive the continuous assessment through an EMA (also contained in the MEmind app, version 1.0). 

All participants will receive their usual treatment (Treatment As Usual, TAU), if they were doing so previously. Participants will be followed-up at 6 months.

### 2.2. Sample

Our study will be composed of three cohorts of participants recruited in different settings. 

#### 2.2.1. Cohort 1: Clinical Setting 

The clinical sample will consist of adolescents with a history of recent suicidal behavior or NSSIs—in the month before recruitment—seen in the emergency department, in the mental health hospitalization unit, or in the mental health outpatient clinics of seven hospitals located in the three Spanish regions of Madrid, Andalusia, and Asturias. 

The inclusion criteria for this group are:

- Being between 13 and 17 years old.
- Presenting a suicide attempt or an NSSI in the month previous to recruitment.
- Being able to understand the project and being interested in participating.
- Having parents or legal guardians capable of signing the informed consent.
- Being fluent in Spanish.
- Owning a smartphone with internet access and iOS or Android operating system.

The exclusion criteria for this group are:

- Refusal to install the mobile application.
- Any circumstance or indication that discourages the regular use of a mobile phone (e.g., addiction to new technologies).

#### 2.2.2. Cohort 2: High School

The high school sample will consist of adolescents aged 13 to 17 from ten secondary schools in the city of Madrid.

The inclusion criteria for this group are:

- Being between 13 and 17 years old.
- Testing positive in the screening that will be carried out at the centers (explained below).
- Being able to understand the project and being interested in participating
- Having parents or legal guardians capable of signing the informed consent.
- Being fluent in Spanish
- Owning a smartphone with internet access and iOS or Android operating system.

The exclusion criteria for this group are:

- Refusal to install the mobile application.
- Any circumstance or indication that discourages the regular use of a mobile phone (e.g., addiction to new technologies).

#### 2.2.3. Cohort 3: University Setting

This sample will consist of university students aged 18 to 24 at the University of Zaragoza.

The inclusion criteria:

- Testing positive in the screening that will be carried out at the center (explained below).
- Being able to understand the project and being interested in participating.
- Being capable of signing the informed consent.
- Being fluent in Spanish.
- Owning a smartphone with internet access and iOS or Android operating system.

The exclusion criteria:

- Refusal to install the mobile application.
- Any circumstance or indication that discourages the regular use of a mobile phone (e.g., addiction to new technologies).

### 2.3. Sample Size Calculation

Sample size calculation will be performed using G*Power software, version 3.1. Based on previous clinical trials that explore the ability of interventions in SI reduction, in the midterm [36] we estimate an effect size of 0.5. Since the alpha error is set at 5% and the power at 95%, performing the modality “Means: Difference from constant (one sample case)”, the program determined that we would need a minimum of 45 participants per branch. Considering our recruitment capacity and assuming dropouts, we set the sample size at 60 participants per branch (120 in total). In order to stratify by center—so that we can explore the possibility of bias by different recruitment centers—we will try to have this sample size in each of the participating hospitals as well as in the educational contexts, which would result in a total sample of 120 × 9 = 1080 participants.

### 2.4. General Procedure:

The study involves three cohorts of participants, each followed for six months, with weekly and monthly telephone check-ins and face-to-face visits at the three-month and six-month marks. These follow-ups are crucial for assessing the intervention’s effectiveness and making any necessary adjustments to the participants’ safety plans.

After enrollment, the app is installed on the participant’s phone, and a psychologist or psychiatrist assists in setting up the app. The participants in the intervention group will be taught how to use the safety plan. 

**Figure 1:**
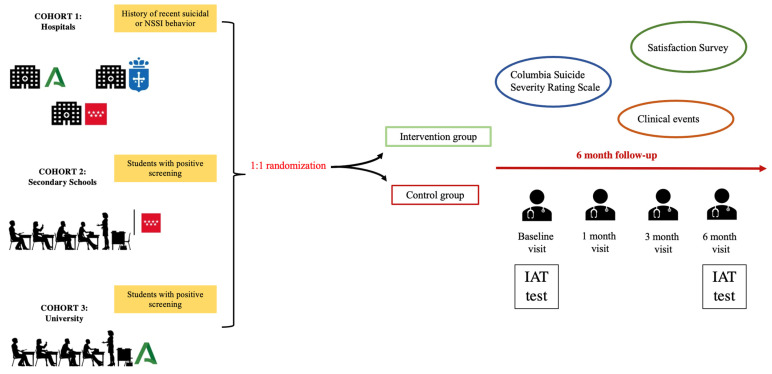
SmartCrisis-Teen study design.

At the end of the six-month period, participants are assessed for changes in suicidal behavior and NSSIs. The follow-up also includes a review of the participants’ use of the app, their interaction with the safety plan and satisfaction with the intervention. Figure 1 illustrate the procedure. 

### 2.5. Procedure: Clinical Setting

Once the recruitment period starts, the referring psychiatrist or psychologist will check the patient’s eligibility and explain the project in detail. If the patient shows interest, an interview will be held with their parents or guardians, who will read and sign the informed consent form. They will be given enough time to study the information sheet and any doubts they may have will be explained. They will also be told that participation is completely voluntary and that they can withdraw their consent at any time. If they wish, they can continue to use the application for free after having finished the study. 

### 2.6. Procedure: High School and University Setting

In the collaborating educational centers, the study will be explained and the informed consent will be obtained for the first phase of the study, which consists of a preliminary screening using the Paykel Suicide Risk Scale [37] and the Inventory of Statements about Self-Injury scale (ISAS) that assess NSSIs [38]. Students who have tested positive will be eligible to participate in the next phase of the study.

### 2.7. Randomization and Follow-Up

A 1:1 randomization will take place after enrollment, where each participant will be assigned to one of the two groups. In both the control and intervention groups, an initial visit will take place during which a psychiatrist or psychologist will install the MEmind app on the participants’ mobile phone so that they can answer the questionnaires contained in the app with the guidance of the evaluator. In the case of self-administered questionnaires, the participants will answer directly on the screen of their mobile phone. In the case of hetero-administered questionnaires, the evaluator will complete them via the MEmind web portal, which is synchronized with the application.

Participants will be followed for six months, with weekly and monthly telephone visits and face-to-face visits at three and six months. In the intervention group, participants will also be given access to the safety plan included in the MEmind application. During the baseline visit, the plan will be set up for the first time and how it works will be explained. 

### 2.8. Psychometric Assessment

The assessments will be carried out by a trained psychologist. The CSSRS [34] will be used to measure suicidal behavior and suicidal ideation. Sociodemographic data will also be collected, and a satisfaction survey will be carried out.

### 2.9. Implicit Association Test

The Spanish versions of the SI-AT and D/S-IAT [39] will be administered. These are 5 min tests administered by computer. The D-S IAT requires participants to match words from the following two semantic fields: “death” (die, dead, deceased, lifeless, and suicide) and “life” (alive, survive, live, thrive, and breathing). The attributes “me” (I, myself, my, self, and mine) and “not me” (they, them, their, theirs, and other) are used to categorize the words. The reaction times are processed using the standard D algorithm for IATs [40]. The algorithm takes into account the reaction time for the implicit association with death (faster reaction scores positively) and the reaction time for the implicit association with life (faster reaction scores negatively). Therefore, a participant with an unconscious bias towards life will have a negative score and a person with an unconscious bias towards death will have a positive score. The greater the score, the stronger the association in one direction or the other. A similar principle is applied for the SI-IAT, albeit using semantic fields related to self-harm. 

### 2.10. Ecological Momentary Assessment 

Digital monitoring will be carried out using the EMA methodology via the MEmind app. This app offers daily short questions that appear on the smartphone screen. Each day, a notification will appear with 2–4 random questions, which will appear at a random time during the day (from 9 a.m. to 9 p.m.) and which are part of a pool of 33 questions that make up the questionnaire. 

This questionnaire is based on the Salzburg Suicide Process Questionnaire (SSPQ) [41], the Patient Health Questionnaire-9 (PHQ-9) [42], the Positive and Negative Affect Schedule (PANA) [43], the Interpersonal Needs Questionnaire (INQ) [44], and previous EMA studies [45,46,47,48]. 

Questions will remain as a notification on the screen until they are answered. The application also has a free text field in which the participant can write comments on their status.

The EMA questions belong to six categories:

- Suicidality: 4 questions;
- Non-suicidal self-injury: 2 questions;
- Affect: 9 questions;
- Interpersonal experiences: 11 questions;
- Sleep: 4 questions;
- Apetite and eating: 3 questions.

The complete list of Ecological Momentary Assessment (EMA) questions can be found in Appendix A.

The digital safety plan is incorporated into the MEmind app and consists of a set of personalized coping strategies that the participant can use in the event of a suicidal crisis [20]. The original safety plan was designed by Stanley and Brown (2012) [20] and has been proven in several studies to decrease the risk of suicide [21]. 

It should be designed with the help of clinical staff. The safety plan will take advantage of smartphones by offering participants the ability to call loved ones, show relaxation videos, activate pre-recorded messages, link to websites with health resources, or connect the participant to emergency resources. During the first visit, the safety plan will be customized with the participant based on their personal preferences (e.g., by providing a list of emergency contacts or programming the app to trigger a song, image or video pre-selected by the participant).

The tabs of the safety plan are: 

The security plan consists of 7 tabs. In each tab, the patient can add text messages, voice messages, audios, photos, videos and links to websites.

**Figure 2:**
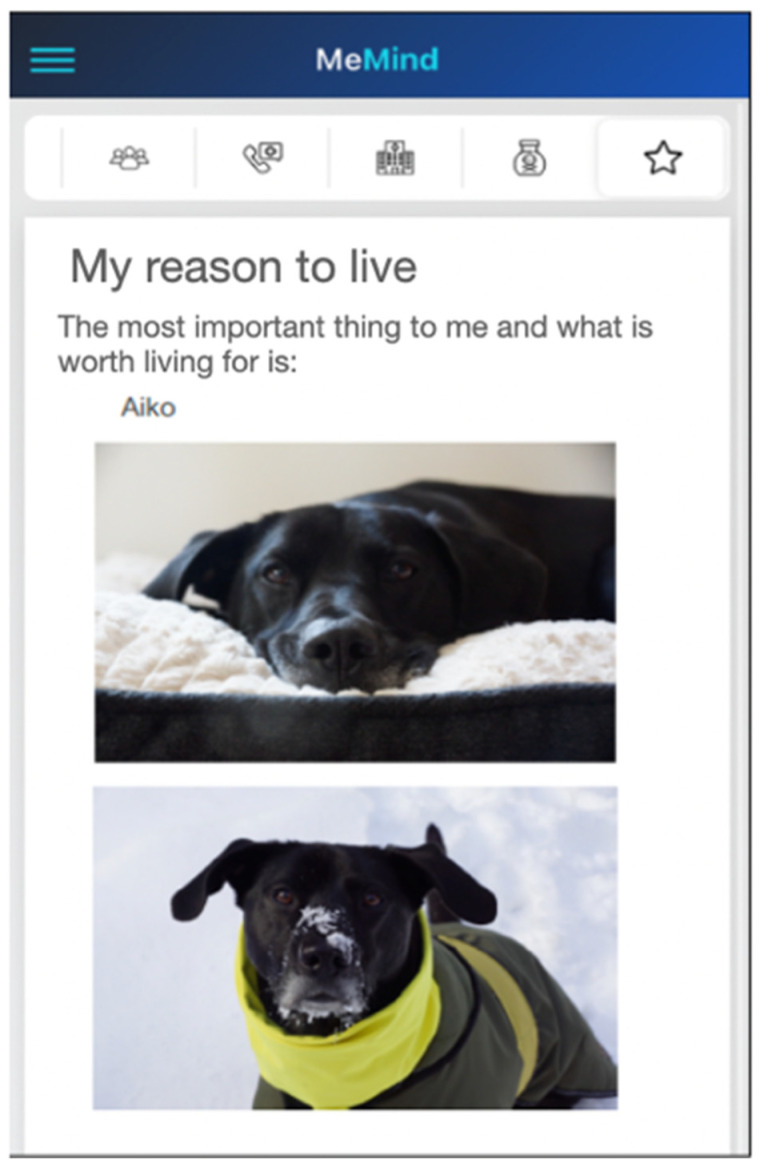
Example of customization of the “Reasons for living” tab.

The 7 tabs in the security plan are as follows:

1. Warning Signs—warning signs (thoughts, images, moods, situations, behaviors, etc.…) that may indicate a crisis is occurring.

2. Internal coping strategies—suggestions for distracting activities that participants can do on their own or without contacting another person (relaxation techniques, physical activityEtc.).

3. External coping strategies—suggestions for distracting activities that can be achieved with the help of the social environment.

4. Personal contacts—list of people in the participant’s support network. Only contacts can be added.

5. Professional contacts—professionals or institutions to contact during a crisis.

6. Safe environment—recommendations for making the environment safe.

7. Reasons for living—an important reason why life is worth living. For example, the participant could include a photograph (see Figure 2).

Apart from configuring the safety plan according to the individual needs of each participant, these tabs can be adapted to both the prevention of a suicide attempt and the prevention of self-harm. Thus, the warning signs can refer to both signs of an impending self-harm crisis and a suicidal crisis, while the safe environment can refer to both lethal-free and sharps-free, depending on the needs of each participant at each point in his or her evolution. The safety plan is multipurpose and can be tailored to each participant’s personal circumstances, providing a customized intervention. In addition, pre-loaded recommendations specific to NSSIs are included in the plan (explained below).

**Figure 3:**
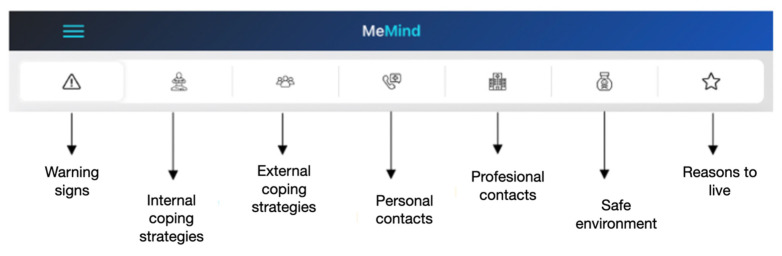
Tabs of the safety plan.

The different tabs contained in the MEmind safety plan are shown in Figure 3. 

Module of recommendations for the management of an NSSI crisis

In addition, the MEmind app includes a set of pre-installed recommendations for managing an NSSI crisis designed for young people. These recommendations include sections on ‘Identifying triggers’, ‘Strategies to use’, ‘Relaxing the five senses’ and ‘Stop’ (a series of intense stimuli that can act as a substitute for self-harm). 

### 2.11. Assessments: Questionnaires 

The assessments will be conducted by psychologists and/or psychiatrists, except for the screening in schools, which will be self-administered and carried out by the participant. All questionnaires will be completed in the MEmind collection portal, which allows for easy completion and immediate uploading to databases. 

The screening will consist of the completion of the following two short scales:

- Paykel Suicide Risk Scale [36];
- Inventory of Statements about Self-Injury scale (ISAS) [37].

The following questionnaires and instruments will be used for the assessment of items related to suicidal behavior and suicide ideation:

- Sociodemographic data including gender, age, family socioeconomic status, grade repetition, sexual orientation, number of siblings (including the participant) and child adoption.
- The Columbia Suicide Severity Rating Scale (CSSRS) [34] will be administered to measure suicide ideation and behavior. This scale will be employed both in the basal and the follow-up appointments.
- In addition, we will verify suicide attempts and suicide deaths through the digital medical record (clinical events).
- The Inventory for Depressive Symptomatology—Clinical (IDSC-30) scale [49] will be administered in order to measure depressive signs and symptoms.
- MacLean Screening Instrument for Borderline Personality Disorder [50].
- The Spanish version of the Death/Suicide Implicit Association Test (D/S-IAT) [38]
- Satisfaction surveys (one qualitative and one quantitative satisfaction survey).

The quantitative survey consists of the following questions, which participants will rate from 1 to 10:

1. Please rate your overall satisfaction with the application.

2. Is the application easy to use? 

3. Is the performance of the application good (speed of navigation, accuracy, lack of errors...)?

4. How do you rate the quality of the graphics?

5. How secure is the application with regard to the processing of personal data?

6. To what extent does the application have a clinical benefit? 

7. Would you be willing to recommend this application to family or friends?

The qualitative survey will ask the participant to comment on how their participation in the project has been, what aspect they liked most, what aspect they liked least and what suggestions they have for improvement, as well as any additional comments they may wish to make. 

Both surveys will be carried out at the six-month visit. 

Finally, participation and retention rates will be calculated.

### 2.12. Assessment Criteria

After six months, a before-and-after comparison will be made in both the control and intervention groups. 

Our main outcome will be a reduction in suicidal behavior, as measured by a score on the CSSRS.

Our secondary outcomes will be as follows:

- The occurrence of suicidal behavior as a clinical event (completed suicide, attempted suicide, ED visit for suicidal ideation, ED visit for self-harm),
- Predictive validity of the D/S IAT and SI IAT tests to clinical events recorded during follow-up.
- Acceptability of the project through user-perceived satisfaction.
- Project feasibility measured by participation and retention.

#### Treatment as Usual (TAU)

Likewise, participants who were previously followed-up will receive their usual treatment (TAU). This will be the psychiatric follow-up (scheduled appointments with the referring psychiatrist/psychologist) in the outpatient clinics for children and adolescents. This approach ensures that the well-being of participants is maintained in accordance with the standards applied to other patients receiving treatment within the national health system.

A continuous quality control phase will be implemented throughout the process to minimize data loss, ensure correct data collection and enable the resolution of any technical issues that may arise with the application. A control panel will be created to facilitate the monitoring of technical incidents, the information collected and the activation of safety plans.

### 2.13. Statistical Analysis

Traditional statistical analyses will be performed using SPSS 25.0 statistical software. For continuous variables, we will use t-tests to compare means between groups and ANOVA for multiple group comparisons. For categorical outcomes, chi-square tests will be employed to examine the frequency distributions.

In order to compare suicidal behavior (CSSRS score) at baseline and at the end of follow-up, a Wilcoxon test will be used. Survival curves (time to a new event, defined as a completed suicide, a suicide attempt or a visit to the emergency room for suicidal ideation) will be calculated using the Kaplan–Meier actuarial method.

To assess the feasibility of the project, the participation rate and retention (by percentage and by survival curve using drop-out as an event) will be calculated. To assess the acceptability of the project, descriptive statistics will be provided for the quantitative satisfaction survey and a content analysis will be conducted for the qualitative satisfaction survey. Binary logistic regression will be used to explore the correlation between the study variables, obtaining crude ORs adjusted for age and sex. 

Additionally, to reduce the probability of bias, we will factor by certain variables that could potentially act as confounders, such as the presence of other health apps in the users’ phones and response latency (which will be measured). 

All tests will be two-tailed, with a significance level of *p* < 0.05 and 95% confidence intervals.

## 3. Limitations

This study incorporates several methodologies to address potential biases, though inherent limitations remain. The digital nature of our intervention may introduce skewness when compared to traditional clinical interviews. We plan to mitigate this by employing statistical controls such as covariance analysis to adjust for baseline differences. 

Furthermore, while randomization reduces selection bias, the possibility of non-random dropout exists, which could lead to attrition bias. We will employ intention-to-treat analysis to minimize the effects of dropout. Despite these efforts, some external factors influencing participant engagement with the digital tool may not be fully controllable. We acknowledge these limitations and will discuss their potential impact on our findings, aiming to provide a transparent and critical assessment of our study’s results.

## 4. Expected Results

This project is highly feasible, as the mobile application it uses has already been tested in previous studies in the adult population [35]. With this intervention, we hope to contribute to the mental health of the adolescent population in the following two ways: Firstly, by decreasing suicidal behavior over the course of follow-up, i.e., both suicidal ideation and suicide attempts, which will be reflected in lower CSSRS scores in the intervention group. Secondly, by reducing NSSIs through the acquisition of coping strategies that can be used in times of risk. This training should be a useful tool beyond the follow-up period of the study and could be applied in the long term. As NSSIs increase the risk of a subsequent suicide attempt, it is hoped that reducing NSSI episodes will also reduce suicide risk.

We also expect that The D/S IAT and SI IAT will demonstrate predictive validity with respect to the gold standard CSSRS.

Finally, we also expect the project to be well accepted by the participants, which will be reflected in the satisfaction surveys provided. Demonstrating its feasibility will allow it to be implemented on a large scale.

Monitoring will be a source of information for understanding suicidal behavior, NSSIs and their associated factors. Real-time monitoring will facilitate an individualized approach that will allow for the design of prevention strategies tailored to the uniqueness of each patient. 

Finally, it is important to stress the importance of transmitting to the adolescent population the need to seek help if they find themselves in a situation of suicidal risk. Given that this is a population that integrates mobile devices into their daily lives, it is expected that the demand for help will be more accessible; it is also hoped that the reception of the preventive approach will be improved.

## 5. Conclusions

Suicidal behavior and NSSIs are a major health problem in the adolescent population. Our intervention could be an effective digital tool in times of suicidal crisis, which complements the traditional clinical approach. It is also a cost-effective intervention which facilitates its implementation on a large scale. This is particularly interesting in the adolescent population, which is characterized by its high use of and competence with new technologies. Moreover, the possibility of customizing and personalizing the digital safety plan makes it an individually tailored intervention adapted to the needs of each participant.

This project will evaluate the effectiveness of a digital tool for the prevention of suicidal and self-harming behavior, which we hope will be of great use in dealing with adolescents’ moments of crisis. Our platform is easily applicable to the health care environment; the application is free for the user and is relatively self-sufficient. In conclusion, our project is designed not only to support adolescents in immediate crisis but also to foster longer-term resilience by equipping them with strategies to manage their mental health proactively. Finally, it is eminently empirical and easily extendable to various fields, and it can become a useful tool of great social value to improve the mental health of adolescents. 

## Data Availability

Data not available yet.

## Appendix A. Ecological Momentary Assessment (EMA) Questionnaire

**Category**	**Variable**	**Question**	**Minimum Value**	**Maximum Value**	**Scoring**
Suicidality (4 questions)	Pasive SI	My wish to live is	No wish to live	Maximum wish lo live	1–7
		My wish to die is	No wish to die	Maximum wish to die	1–7
		Do you have thoughts of hurting yourself in some way?	Not at all	Nearly every day	0–3
	Active SI	Are you able to keep yourself safe right now?	I definitely can keep myself safe	I definitely cannot keep myself safe	1–5
Non-Suicidal Self-Injuries (2 questions)	NSSIs	At any point in the last 24 h, did you harm yourself on purpose?	Yes	No	Yes/No
		Since the last prompt, have you felt an urge or wanted to harm or injure yourself on purpose?	Not at all	Extremely	1–5
Affect (9 questions)	Psychological pain	I feel psychological pain	No pain	Maximum pain	1–7
	Stress	I feel stressed out today (with pressure, overwhelmed)	No stress	Maximum stress	1–7
	Restlessness	I feel restless (agitates), with the need to keep moving	No restlessness	Maximum restlessness	1–7
	Hopelessness	I feel full of hope	No hope	Maximum hope	1–7
	Self-hatred	I feel hatred or anger towards myself	No hatred	Maximum hatred	1–7
	Hatred of others	I feel hatred or anger towards others	No hatred	Maximum hatred	1–7
	Anxiety	At this moment I feel nervous	Very slightly or not at all	Extremely	1–5
	Sadness	At this moment I feel sad	Very slightly or not at all	Extremely	1–5
	Happiness	At this moment I feel happy	Very slightly or not at all	Extremely	1–5
Interpersonal experiences (11 questions)	Thwarted belongingness	I wish there was a trusted person with whom I can talk about all my personal issues	Not at all	Absolutely	1–7
		I feel like an outsider	Not at all	Absolutely	1–7
	Lack of recognition	I wish I received more recognition and love from others	Not at all	Absolutely	1–7
	Lack of independence	I have the impression that important people around me want to decide for me what I should think and do	Not at all	Absolutely	1–7
	Criticism	Since the last prompt have you felt insulted or criticized?	Not at all	Extremely	1–5
	Thwarted belongingness	Since the last prompt have you felt rejected, abandoned, excluded or left out?	Not at all	Extremely	1–5
	Perceived burdensomeness	I believe I am contributing to the well-being of my family/friends	Not at all	Absolutely	1–7
		believe I am contributing to the well-being of the people around me	Not at all	Absolutely	1–7
		I feel disconnected from other people	Not at all	Absolutely	1–7
		I feel like a burden to others	Not at all true for me	Very true for me	1–7
		I feel useless	Not at all true for me	Very true for me	1–7
Sleep (4 questions)	Sleep maintenance	Last night I had trouble staying sleep	None	Very severe	0–4
	Sleep-derived quality of life	Others think that sleep problems affect my quality of life	Not at all	Absolutely	0–4
	Sleep dissatisfaction	Today I am satisfied with my sleep	Very unsatisfied	Very satisfied	0–4
	Sleep-derived interference with daily activity	My sleep problems are interfering with my daily activity	Not at all	Very much	0–4
Appetite and eating (3 questions)	Appetite	In the last few days, I have been hungry	Never	All the time	0–4
	Taste	In the last days when I eat, the food tastes	Very bad	Very good	0–4
	Number of meals	In the last few days, I usually do	Less than one meal a day	More thn three meals a day	0–4
